# TROP2 in colorectal carcinoma: associations with histopathology, molecular phenotype, and patient prognosis

**DOI:** 10.1002/2056-4538.12394

**Published:** 2024-08-23

**Authors:** Sebastian Foersch, Maxime Schmitt, Anne‐Sophie Litmeyer, Markus Tschurtschenthaler, Thomas Gress, Detlef K Bartsch, Nicole Pfarr, Katja Steiger, Carsten Denkert, Moritz Jesinghaus

**Affiliations:** ^1^ Institute of Pathology University Medical Center Mainz Germany; ^2^ Institute of Pathology Philipps‐University Marburg und University Hospital Marburg Marburg Germany; ^3^ Internal Medicine II, Klinikum rechts der Isar Technical University Munich Munich Germany; ^4^ Institute for Translational Cancer Research, German Cancer Consortium (DKTK), Partner Site Munich Munich Germany; ^5^ Department of Gastroenterology, Endocrinology and Infectious Diseases Philipps‐University Marburg and University Hospital Marburg Marburg Germany; ^6^ Department of Surgery Philipps‐University Marburg and University Hospital Marburg Marburg Germany; ^7^ Institute of Pathology Technical University of Munich Munich Germany

**Keywords:** TROP2, colorectal carcinoma, prognosis

## Abstract

Antibody–drug conjugates (ADCs) directed to trophoblast cell surface antigen 2 (TROP2) have gained approval as a therapeutic option for advanced triple‐negative breast cancer, and TROP2 expression has been linked to unfavourable outcomes in various malignancies. In colorectal carcinoma (CRC), there is still a lack of comprehensive studies on its expression frequency and its prognostic implications in relation to the main clinicopathological parameters. We examined the expression of TROP2 in a large cohort of 1,052 CRC cases and correlated our findings with histopathological and molecular parameters, tumour stage, and patient outcomes. TROP2 was heterogeneously expressed in 214/1,052 CRCs (20.3%), with only a fraction of strongly positive tumours. TROP2 expression significantly correlated with an invasive histological phenotype (e.g. increased tumour budding/aggressive histopathological subtypes), advanced tumour stage, microsatellite stable tumours, and p53 alterations. While TROP2 expression was prognostic in univariable analyses of the overall cohort (e.g. for disease‐free survival, *p* < 0.001), it exhibited distinct variations among important clinicopathological subgroups (e.g. right‐ versus left‐sided CRC, microsatellite stable versus unstable CRC, Union for International Cancer Control [UICC] stages) and lost its significance in multivariable analyses that included stage and CRC histopathology. In summary, TROP2 is quite frequently expressed in CRC and associated with an aggressive histopathological phenotype and microsatellite stable tumours. Future clinical trials investigating anti‐TROP2 ADCs should acknowledge the observed intratumoural heterogeneity, given that only a subset of TROP2‐expressing CRC show strong positivity. The prognostic implications of TROP2 are complex and show substantial variations across crucial clinicopathological subgroups, thus indicating that TROP2 is a suboptimal parameter to predict patient prognosis.

## Introduction

Colorectal carcinoma (CRC) is the third most common cancer in humans concerning incidence and mortality worldwide, with the vast majority of tumour‐related deaths being caused by advanced cancers with metastatic spread [1, 2]. Patients suffering from advanced CRC are usually treated by chemotherapy (usually 5‐fluorouracil with oxaliplatin or irinotecan), which is increasingly complemented by targeted therapy approaches or immunotherapy [3]. However, a significant fraction of patients develop progressive disease and die even under intensive therapy, highlighting the medical need for additional therapeutic options.

Antibody–drug conjugates (ADCs) are a comparatively novel class of pharmaceutical substances, which have recently been approved as therapeutic alternatives for advanced breast, bladder, or gastric cancer [4]. ADCs conjugate cytotoxic agents (payload) to monoclonal antibodies against specific cellular targets via a linker molecule. Sacituzumab govitecan (SG, IMMU‐132, Immunomedics Inc./Gilead Sciences, Morris Plains, NJ, USA) is one of the most promising ADCs, which has been approved as a third‐line therapy for triple‐negative breast cancer (TNBC). SG conjugates SN‐38, an active irinotecan metabolite, to trophoblast cell surface antigen 2 (TROP2) via the peptide‐linker CL2A, which then enables intra‐ and extratumoural release of the drug.

TROP2 is a trans‐membranous protein expressed in a variety of normal tissues (especially trophoblast cells and squamous epithelia) [5, 6] and physiologically acts as a calcium signalling transducer that regulates cell‐growth, migration, and proliferation [7, 8].

TROP2‐positive epithelia have been linked with stem cell properties in normal tissues of several organs [9, 10, 11] and TROP2 expression has been linked with an adverse prognosis in a variety of cancers [12, 13, 14]. For CRC, a recent study investigated TROP2 expression in metastatic CRC and demonstrated prognostic relevance in this subgroup [15]. However, the association and the prognostic value of TROP2 in comparison to conventional histopathological parameters (tumour budding, tumour grade, histopathological subtypes) is still poorly understood and has not yet been comprehensively studied in large CRC collectives.

Therefore, considering the important role of TROP2 in the pharmacodynamics of SG [16, 17, 18, 19], our study investigated TROP2 expression according to the immunoreactive score (IRS) [20, 21, 22] in a large cohort of 1,052 resected CRC. We investigated possible associations with conventional histomorphological parameters of CRC from the World Health Organization (WHO) classification on haematoxylin and eosin (H&E)‐stained slides (tumour budding, WHO grade, histopathological subtypes) [23, 24] as well as important clinicopathological features (tumour location, vascular invasion, Union for International Cancer Control (UICC) stage, microsatellite/p53 status) and finally probed the prognostic value of TROP2 alone and in comparison to the aforementioned parameters.

## Materials and methods

### Cohort characteristics

Our cohort comprised 1,052 CRCs, which were surgically resected between 1997 and 2022 at the University Hospital Rechts der Isar of the Technical University Munich (*n* = 1,042) or the University Hospital Marburg (*n* = 10). Patients with other neoplasms of the colorectal system (e.g. well‐differentiated neuroendocrine tumours and non‐epithelial tumours), appendiceal tumours, incomplete clinicopathological/survival data, or insufficient tissue were excluded.

The median patient age was 69 years. Six‐hundred seven patients were male (*n* = 57.7%). Five‐hundred eight CRCs were right‐sided (caecum to splenic flexure; 48.3%), 426 left‐sided (descending colon and sigmoid colon; 40.5%), and 118 patients suffered from rectal cancers (11.2%). Using the eighth edition of the TNM classification of malignant tumours [25], the distribution of pTNM stages of the cohort cases was as follows: 213 (20.2%) stage I, 351 (33.4%) stage II, 325 (30.9%) stage III, and 163 (15%) stage IV cancers. A relapse was noted for 336 patients (31.9%) and 416 patients (40.7%) died during follow‐up. Tumour‐specific death was recorded for 306 patients (29.1%) (cohort details: supplementary material, Table S1).

The respective clinicopathological characteristics and survival data were extracted from hospital records and from the Munich Cancer Registry. For overall survival (OS), all recorded patient deaths were noted, while only deaths that were declared as tumour associated by the treating clinicians were recorded as events for disease‐specific survival (DSS). For disease‐free survival (DFS), events were defined as either loco‐regional or distant recurrence. Endpoints for all survival comparisons were either events or a loss of follow‐up, in which case the patients were censored at the time of the last available entry. Patients with no event after 120 months were also censored. The treatment concepts of included patients followed internal policies, which were based on the given German guidelines at the time of diagnosis, generally meaning that all patients were intended to receive stage‐adapted treatment.

The study was approved by the local ethic committees of the Technical University of Munich (reference number: 252/16 s) and of the University Hospital Marburg (reference number: AZ 23/21) [24].

### Histopathological characterisation

All neoplasms were classified on H&E‐stained whole tissue sections with reference to the criteria given by the fifth edition of the World Health Organization Classification of Digestive System Tumours (WHO classification [23, 24]). As described previously, this basal characterisation included histopathological subtype, tumour grading (WHO‐grade: low‐grade versus high‐grade), tumour budding activity [Bd1: 0–4 buds, Bd2: 5–9 buds, Bd3: 10 or more buds; evaluated in 20× hotspot according to the International Tumour Budding Consensus Criteria (ITBCC)], lymphatic‐ or venous invasion as well as resection status. Microsatellite status was assessed via MLH1, MSH2, MSH6, and PMS2 immunohistochemistry, as described previously [24, 26, 27, 28]. Assessment of the p53 status was performed via p53 immunohistochemistry (clone DO‐7, DAKO/Agilent, Santa Clara, CA, USA). According to current recommendations, tumours with strong nuclear overexpression of p53 in more than 80% of the tumour or tumours with complete loss of nuclear expression were labelled as aberrant staining indicative of a *TP53* mutation, while heterogeneous expression with mixed intensity was considered as p53 wild‐type staining [29, 30, 31].

### Immunohistochemical analyses of TROP2 expression

**Figure 1:**
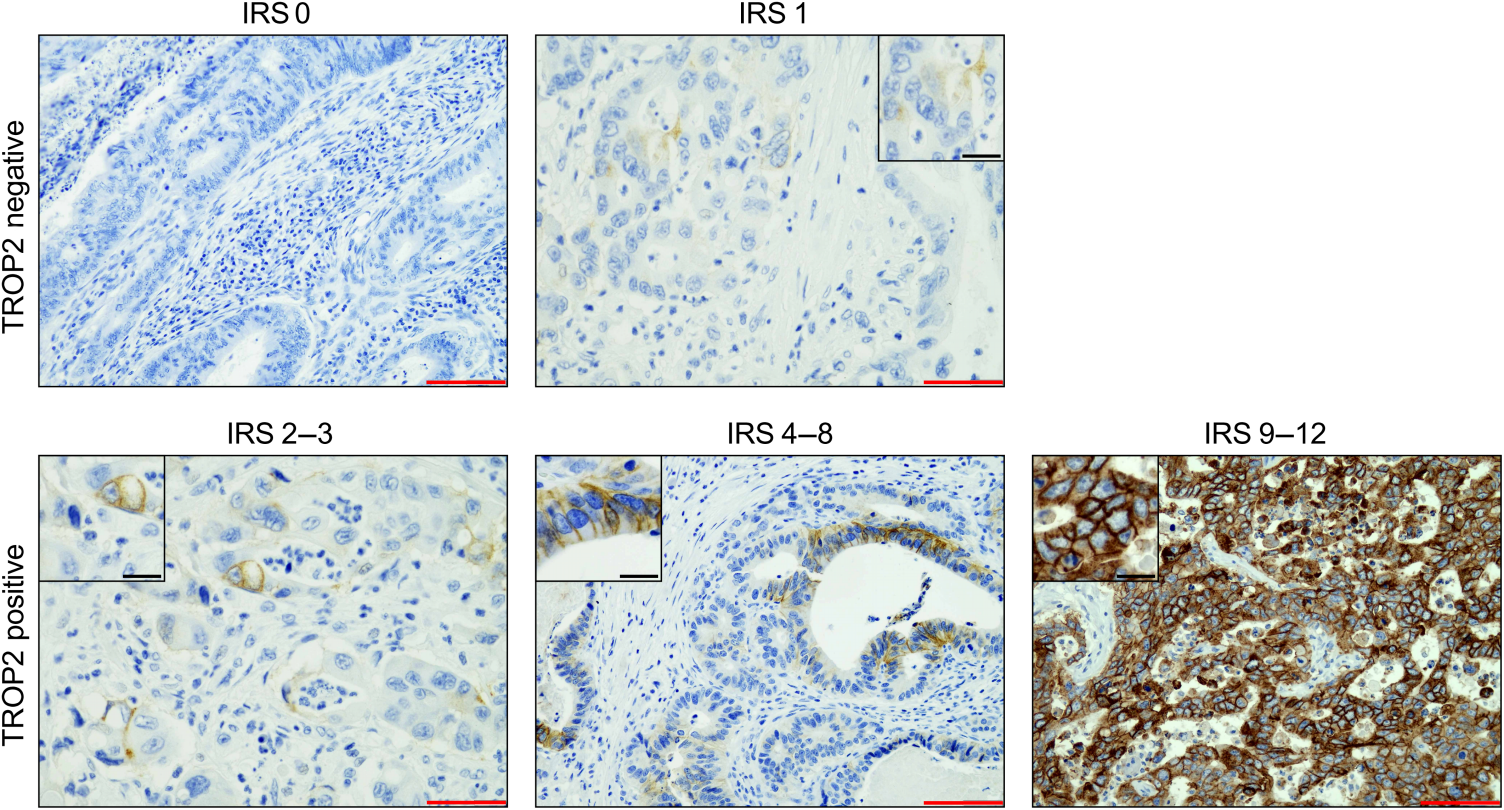
Illustration of TROP2 expression groups. The top panels show TROP2 negative tumours which were either completely negative (IRS 0, left) or showed only very weak, non‐specific staining in single cells (IRS 1, right). The lower panels show TROP2‐expressing CRCs ranging from tumours with medium to strong intensity in <10% of the cells (IRS 2–3, left), tumours with intermediate expression (IRS 4–8, middle), or tumours with strong expression (IRS 9–12, right) (see also Table 1). All tumours with an IRS ≥ 2 were considered TROP2 positive.

Tissue microarrays (TMAs) containing two separate cores from the tumour centre and from the invasive front from 1,052 CRCs were stained with a TROP2 antibody (clone SP 295, dilution 1:600, Abcam, Cambridge, UK) on a LINK48 autostainer (Agilent). TROP2 staining was manually evaluated by an experienced gastrointestinal (GI)‐pathologist (MJ); only membranous staining was considered specific and tonsillar squamous cell epithelium served as an external control. The number of positive carcinoma cells was assessed for each individual patient, counting a minimum of 500 tumour cells, resulting in a cumulative percentage score for both cores that were assigned for each CRC (range: 0–100%). The expression intensity was graded into strong (comparable to normal squamous epithelium), moderate (clearly visible staining but notably weaker), weak (barely perceptible and only notable at high magnifications), and negative (no staining reaction). Afterwards, all carcinomas were assigned to different TROP2 expression groups according to their IRS, which is derived from a sum score of the percentage of expressing cells (score 0–4) and the maximum staining intensity (score 0–3), which are then multiplied by each other. According to the IRS, four TROP2 expression groups were defined (TROP2 negative: IRS 0–1; TROP2 low: IRS 2–3; TROP2 moderate: IRS 4–8; and TROP2 high: IRS 9–12). The detailed algorithm for the determination of the IRS is given in Table 1, and an example of the different IRS is given in Figure 1. TROP2 expression according to the IRS as assessed on the TMA was also correlated with the expression on whole tissue sections in 50 cases. To test the interobserver reproducibility, 100 cases on the TMA were scored by a second pathologist (SF) blinded to the initial evaluation of the main observer.

**Table 1:** Algorithm to determine the IRS

Immunoreactive score	TROP2	
Score	Staining intensity	Percentage of positive cells
0	No staining reaction	0%
1	Weak staining reaction	<10%
2	Moderate staining reaction	10–50%
3	Strong staining reaction	51–80%
4		>80%
**IRS = score (staining intensity) x score (percentage of positive cells)**		
TROP2 expression groups		
IRS 0–1	TROP2 negative	
IRS 2–3	TROP2 low	
IRS 4–8	TROP2 moderate	
IRS 9–12	TROP2 high	

### Statistics

Statistical analyses were performed with SPSS version 28 (SPSS Institute, Chicago, IL, USA) using *χ* ^2^ test as well as *χ* ^2^ test for trends and Fisher's exact test (two‐sided). If necessary, the Bonferroni method was used to correct for multiple testing [32]. Interobserver variance was tested using the spearman‐correlation method. The Cutoff Finder, a publicly available biostatistical tool that represents a bundle of optimisation and visualisation methods for cutoff determination, was used to define optimal cutoffs [33]. Univariable survival probabilities were estimated with the Kaplan–Meier method and log‐rank tests were used to probe the statistical significance of differences. Mean and median survival is presented with 95% CIs. Hazard ratios (HRs) for univariable survival analyses were determined using the univariate Cox proportional hazards regression model. Multivariable survival analysis was performed with the Cox proportional hazards model and respective effect estimates of the HR are presented with 95% CIs. Interobserver variability was analysed by using *κ*‐statistics; the interpretation of kappa values was guided by the classification proposed by Landis and Koch [34] (*κ* < 0: less than chance agreement, *κ* = 0.01–0.20: slight agreement, *κ* = 0.21–0.40: fair agreement, *κ* = 0.41–0.60: moderate agreement, *κ* = 0.61–0.80: substantial agreement, *κ* = 0.81–0.99: almost perfect agreement). All statistical tests were performed two‐sided; *p* values of ≤0.05 were considered significant.

## Results

### Prognostic impact of histomorphological factors, staging parameters, and molecular alterations in univariable survival analyses

As described previously and as depicted in detail in supplementary material, Table S1, all of the conventional H&E‐based histomorphological parameters (tumour budding, histopathological subtypes, WHO grade) profoundly impacted all survival parameters (*p* < 0.001 for OS, DSS, DFS, respectively) [24]. TNM status and the resulting UICC stage as well as other known adverse staging parameters such as perineural, lymphatic‐, or venous invasion or positive resection margins were also significantly associated with patient survival (*p* < 0.001 for OS, DSS, and DFS, respectively). Patients with microsatellite instability (MSI)‐high CRC showed a more favourable outcome compared with microsatellite stable (MSS) patients, whereas abnormal expression of p53 was associated with a worse DSS and DFS, but showed no impact on OS.

### Frequency and subgrouping of TROP2 expression

**Figure 2:**
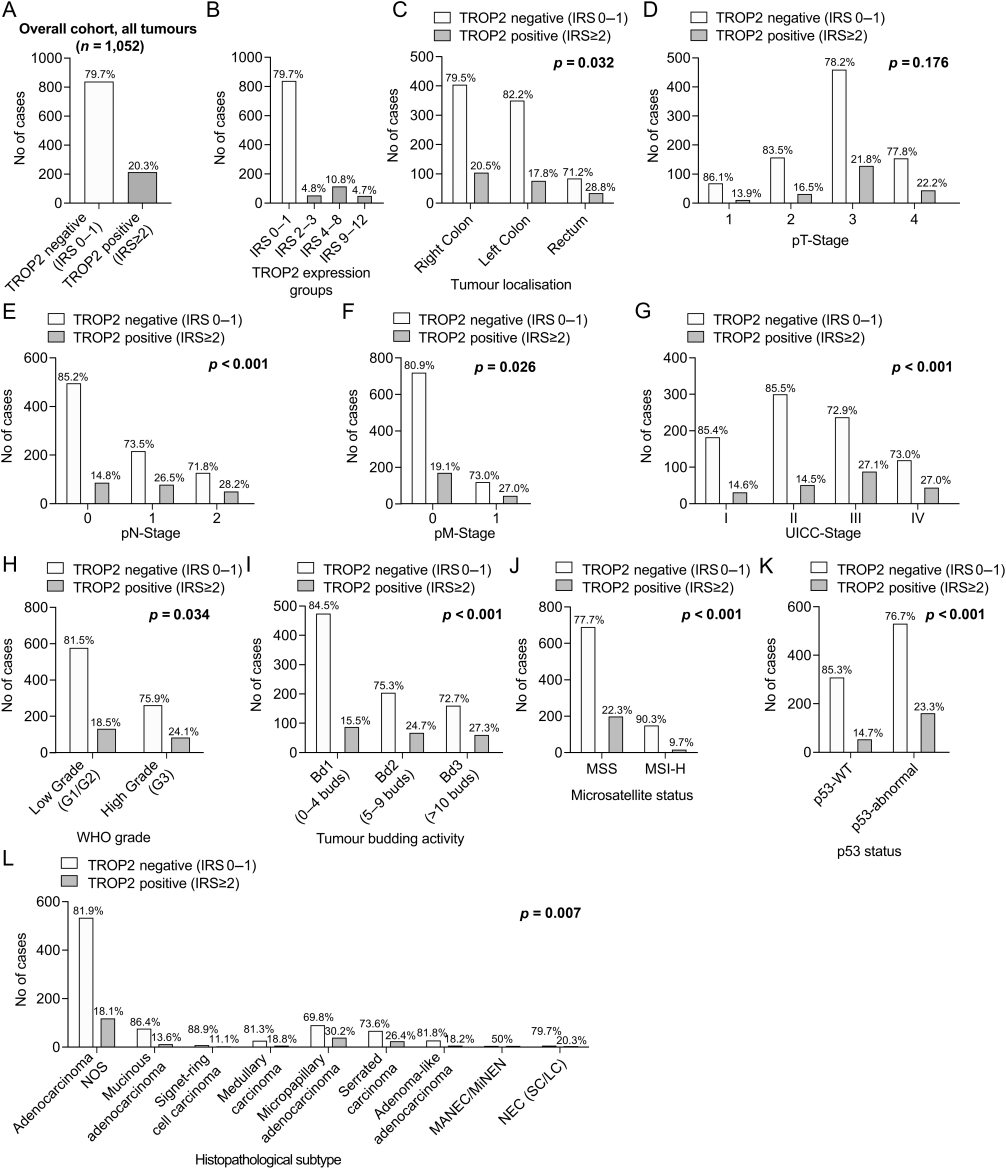
Frequency and clinicopathological associations of TROP2 expression. (A) Frequency of TROP2‐expressing CRC in the overall cohort. (B) Frequency of the different degrees of TROP2 expression in the overall cohort. (C) Association of TROP2 expression with tumour location showing a slight enrichment in rectal cancers. (D–G) Association of TROP2 with pT, pN, and pM stage as well as the resulting UICC stage. (H) Association of TROP2 with tumour grade according to the WHO grading system. (I) Association of tumour budding activity according to the International Tumour Budding Consensus Subgroups [26]. (J, K) Association of TROP2 with microsatellite status and immunohistochemical p53 expression. (L) Frequency of TROP2 expression among histopathological CRC subtypes (WHO classification).

As depicted in Figure 2 and supplementary material, Table S2, 214 carcinomas showed TROP2 expression (IRS ≥ 2, 20.3%), whereas 838 carcinomas were entirely negative or showed very weak expression in single cells (IRS 0–1, 79.7%) and were, therefore, labelled as TROP2 negative. According to the IRS, 51 CRCs showed low expression of TROP2 (IRS 2–3, 4.8%), 114 neoplasms showed moderate expression of TROP2 (IRS 4–8, 10.8%), and 49 carcinomas showed strong expression of TROP2 (IRS 9–12, 4.7%). After initial statistical subgrouping using the Cutoff Finder [33], we decided to group all carcinomas with any significant TROP2 expression (IRS ≥ 2) as TROP2 positive, as we detected no statistical differences regarding patient survival between the different TROP2 expression grades in our initial univariable survival analyses. Interobserver analyses of 100 separately investigated cases showed excellent interobserver reproducibility (*κ* = 0.91). There was high concordance (*κ* = 0.78) between the TMA and the respective whole tissue sections.

### TROP2 expression is associated with metastatic spread and advanced tumour stage

CRCs with TROP2 expression were significantly associated with nodal positive cases (*p* < 0.001) and those with distant metastases (*p* = 0.026). Consequently, we observed a highly significant enrichment of TROP2‐expressing cases in higher UICC stages (*p* < 0.001, Figure 2 and supplementary material, Table S2). Furthermore, TROP2 expression was significantly more frequent in cases that showed invasion of lymphatic (*p* < 0.001) and/or blood vessels (*p* = 0.021). No association was noted with local tumour extension (pT‐stage, *p* = 0.176). While we observed no statistical differences regarding TROP2 expression between right‐ and left‐sided cancers, we observed a slightly higher rate of TROP2 positivity in rectal cancers (*p* = 0.032, Figure 2C–G).

### CRCs with adverse histopathological factors show a significantly higher frequency of TROP2 expression

As depicted in Figure 2 and supplementary material, Table S2, TROP2‐expressing CRCs were significantly associated with histopathological features that are attributed with an aggressive clinical behaviour [24, 26, 35]. Cases that showed an increased (Bd2) or even high level (Bd3) of tumour budding according to the ITBCC scoring system showed a much higher frequency of TROP2 positivity than carcinomas with no or low tumour budding (Bd1, *p* < 0.001). Complementary to this, clinically aggressive histopathological CRC subtypes such as the micropapillary variant of CRC or colorectal mixed adenoneuroendocrine carcinoma/neuroendocrine carcinoma (NEC) were significantly more likely to express TROP2 compared with adenocarcinomas not otherwise specified or less aggressive subtypes such as medullary‐ or adenoma‐like adenocarcinomas (*p* = 0.007), as were poorly differentiated carcinomas according to the WHO grade (*p* = 0.034, Figure 2H–L).

### TROP2 expression is highly enriched in MSS and TP53‐mutated CRC

We observed highly significant differences regarding the frequency of TROP2‐positive CRCs between MSS (22.3%) and microsatellite unstable CRCs (9.7%), as TROP2 expression was significantly more frequent in MSS carcinomas (*p* < 0.001) and comparatively rare in microsatellite unstable CRC (Figure 2). Consistent with this observation, TROP2 expression was also significantly more common in those CRCs that had an abnormal p53 expression profile indicative of a *TP53* alteration (*p* < 0.001).

### TROP2 expression is associated with poor survival in univariable but not in multivariable analyses of the overall cohort

In univariable survival analyses (log‐rank test, univariable Cox regression) in the overall cohort, CRCs with expression of TROP2 were associated with significantly shortened OS (*p* = 0.011, HR = 1.345), DSS (*p* = 0.007, HR = 1.432), and DFS (*p* < 0.001, HR = 1.638). For DFS, patients with TROP2‐expressing CRCs had a mean DFS of 69.25 months compared with 85.42 months for TROP2‐negative carcinomas. In multivariable Cox regression analyses incorporating sex, age, UICC stage, tumour location (right‐ versus left‐sided), conventional histopathological parameters (tumour budding, WHO grade, and histopathological subtype), presence/absence of lymphatic/blood vessel invasion, resection status as well as p53 and MSI status, TROP2 expression was not an independent prognostic factor (OS: HR: 1.09, *p* = 0.45, data not shown; DSS: HR: 1.04, *p* = 0.73, data not shown; and DFS: HR: 1.19, *p* = 0.167, Table 2).

**Table 2:** Multivariable survival analyses (disease‐free survival) of TROP2 expression in the whole cohort including tumour stage, tumour location as well as histopathological and molecular parameters

	HR (DFS)	Lower CI (95%)	Upper CI (95%)	*p* value
TROP2 expression				
TROP2 negative	1.00			0.168
TROP2 positive	1.20	0.93	1.55	
Tumour budding activity				
Bd1 (no/low tumour budding)	1.00			<0.001
Bd2 (intermediate tumour budding)	3.38	2.44	4.68	
Bd3 (high tumour budding)	6.44	4.49	9.24	
Histopathological Subtype				
Adenocarcinoma NOS	1.00			0.071
Mucinous adenocarcinoma	1.06	0.69	1.64	
Signet‐ring cell carcinoma	1.20	0.47	3.05	
Medullary carcinoma	0.26	0.06	1.12	
Micropapillary adenocarcinoma	0.81	0.60	1.10	
Serrated carcinoma	1.11	0.73	1.70	
Adenoma‐like adenocarcinoma	0.30	0.04	2.19	
MANEC/MiNEN	1.12	0.51	2.46	
NEC (SC/LC)	0.57	0.18	1.85	
UICC stage				
I	1.00			0.001
II	1.30	0.81	2.09	
III	1.35	0.76	2.40	
IV	2.65	1.49	4.70	
Lymphangiosis				
L0	1.00			0.094
L1	1.44	0.94	2.21	
Blood vessel invasion				
V0	1.00			0.189
V1	1.24	0.90	1.71	
Perineural invasion				
Pn0	1.00			0.226
Pn1	1.24	0.87	1.76	
Resection status				
R0	1.00			0.004
R1	1.75	1.19	2.56	
R2	1.04	0.65	1.67	
WHO grade				
Low‐grade (formerly G1/G2)	1.00			0.285
High‐grade (formerly G3)	1.15	0.89	1.47	
Microsatellite status				
Microsatellite stable	1.00			0.642
Microsatellite unstable	1.11	0.71	1.76	
p53 status				
Wild‐type	1.00			0.689
Aberrant	1.06	0.80	1.41	
Tumour localisation				
Right‐sided colon	1.00			0.569
Left‐sided colon/rectum	1.07	0.85	1.35	
Gender				
Female	1.00			0.896
Male	1.02	0.81	1.27	
Age				
Below median	1.00			0.576
Median and above	1.07	0.85	1.34	

MANEC, mixed adenoneuroendocrine carcinoma; NOS, not otherwise specified.

### Prognostic relevance of TROP2 expression in right‐ and left‐sided colorectal cancer, UICC stages, and molecular subgroups

**Figure 3:**
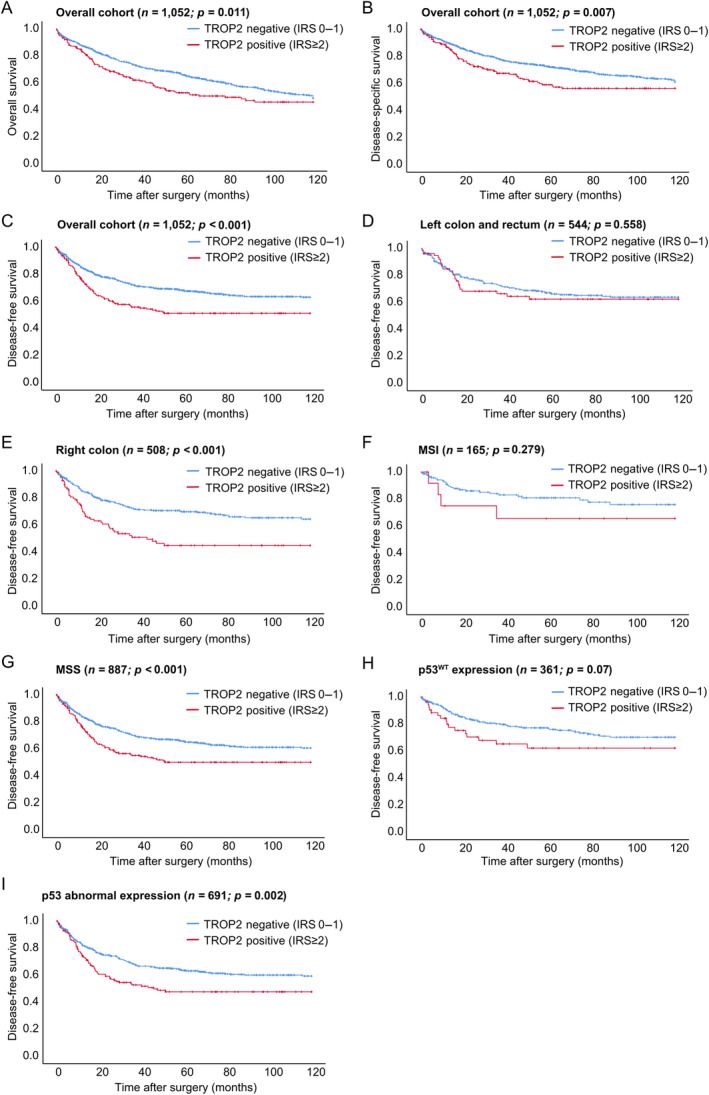
Univariable survival analyses regarding the prognostic relevance of TROP2 expression in the overall cohort and in specific clinicopathological subgroups. (A–C) Univariable survival analysis (log‐rank test) of TROP2 expression in association with overall, disease‐specific, and disease‐free survival. (D) Univariable survival analysis (log‐rank test) of TROP2 expression in association with disease‐free survival in the subgroup of left‐sided and rectal cancers. (E) Univariable survival analysis (log‐rank test) of TROP2 expression in association with disease‐free survival in the subgroup of right‐sided cancers. (F) Univariable survival analysis (log‐rank test) of TROP2 expression in association with disease‐free survival in the subgroup of microsatellite unstable (MSI‐high) CRC. (G) Univariable survival analysis (log‐rank test) of TROP2 expression in association with disease‐free survival in the subgroup of microsatellite stable (MSS) CRC. (H) Univariable survival analysis (log‐rank test) of TROP2 expression in association with disease‐free survival in CRC with p53^WT^ (wild‐type) expression. (I) Univariable survival analysis (log‐rank test) of TROP2 expression in association with disease‐free survival in CRC with abnormal p53 expression.

We observed differing results regarding the prognostic relevance of TROP2 expression in univariable analyses between right‐ and left‐sided tumours. In right‐sided CRC, TROP2 expression was associated with impaired OS (*p* = 0.05, HR = 1.37), DSS (*p* = 0.014, HR = 1.58), and DFS (*p* < 0.001, HR = 2.03, Figure 3), whereas no survival differences were observed in left‐sided cancers (OS, DSS, DFS: *p* > 0.05, respectively). In multivariable analyses of right‐sided CRC including the variables mentioned above, the prognostic value of TROP2 expression was not maintained (OS, HR: 1.09, *p* = 0.45, data not shown; DSS: HR: 1.04, *p* = 0.73, data not shown; DFS: HR: 1.19, *p* = 0.16, supplementary material, Table S3).

Separate univariable analyses between the four UICC stages revealed that TROP2 expression was solely prognostically relevant in UICC stage III cancers (OS: *p* = 0.04, HR = 1.57; DSS: *p* = 0.02, HR = 1.55, DFS: *p* = 0.02, HR = 1.66), but not in stage I, II, or IV CRC (*p* = n.s., see supplementary material, Figure S1 for DSS and supplementary material, Figure S2 for OS) or in multivariable analyses.

In separate univariable survival analyses between MSS and microsatellite unstable CRC, TROP2 expression was only prognostically significant in MSS CRC (OS: *p* = 0.05, HR = 1.37; DSS: *p* = 0.014, HR = 1.58; DFS: *p* < 0.001, HR = 2.03, Figure 3), while no survival association was evident in microsatellite unstable cancers (OS, DSS, DFS: *p* > 0.05, respectively). Similarly, TROP2 was associated with poorer survival in CRCs with abnormal p53 expression (OS: *p* = 0.05, HR = 1.37; DSS: *p* = 0.014, HR = 1.58; DFS: *p* < 0.001, HR = 2.03, Figure 3), but not in those with immunohistochemical wild‐type p53 expression (OS, DSS, DFS: *p* > 0.05, respectively). In multivariable analyses of these subgroups, TROP2 was not an independent prognostic factor.

## Discussion

Expression of the transmembrane glycoprotein TROP2 has been described in a variety of epithelial and non‐epithelial malignancies [8, 12]. TROP2 recently regained the attention of the scientific community after the approval of SG, an ADC targeting TROP2, as a therapeutic alternative for advanced TNBC [36]. In CRC, previous mechanistic studies identified TROP2 as a propagator of tumour proliferation and invasiveness [37, 38, 39, 40], whereas others identified overexpression as an independent prognostic biomarker [15, 38]. Our study assessed TROP2 expression in a substantial cohort of 1,052 primary CRCs, to our knowledge, representing the most extensive CRC collection to have examined TROP2 expression so far. Our emphasis was on its correlation with conventional histopathological factors, tumour stage as well as molecular alterations and to explore the prognostic significance of TROP2 expression in comparison to the aforementioned parameters.

In line with prior mechanistic findings, we noted a robust correlation between invasive histopathological phenotypes and TROP2 expression. CRCs exhibiting an elevated or high degree of tumour budding [24, 26, 41] – an unfavourable histopathological parameter assessing a cancer's capacity for dissociative growth – demonstrated a significantly greater prevalence of TROP2 expression compared with CRCs without dissociative growth. In accordance with the aforementioned, a strong accumulation of TROP2 positive tumours was detected in aggressive histological CRC subtypes such as micropapillary carcinomas or NEC. Conversely, less aggressive variants like adenoma‐like or medullary adenocarcinomas exhibited lower frequencies of TROP2 expression [24, 42, 43, 44, 45]. These histopathological observations align with the inverse correlation of TROP2 expression with generally favourable molecular characteristics. Specifically, CRCs with MSI‐high and wild‐type p53 expression demonstrated significantly lower expression rates compared with MSS tumours [24] and those with abnormal p53 expression [31]. Therefore, our findings indicate a pronounced presence of TROP2 expression in CRCs with unfavourable histomorphological and/or molecular characteristics. This suggests that therapies targeting TROP2 may effectively target these aggressive neoplasms in a precise and focused manner. Nevertheless, it is crucial to recognise the significant intratumoural heterogeneity of TROP2 expression, as strong expression was observed in only about a quarter of all TROP2‐positive CRC cases, which seems to differ from other cancer types such as TNBC [46]. This aspect should be taken into consideration in future clinical trials involving SG for CRC patients, as prior studies included CRC patients without accounting for their TROP2 expression status [17].

Having noted the robust link between TROP2 expression and unfavourable clinicopathological factors, we then investigated its prognostic implications. Consistent with earlier findings, univariable survival analyses conducted on our cohort of over 1,000 CRC cases revealed remarkably diminished OS, DSS, and DFS in patients with tumours expressing TROP2 compared with those with TROP2 negative neoplasms. However, in subsequent survival analyses of crucial clinicopathological subgroups, noteworthy variations emerged in the prognostic significance of TROP2 based on tumour location and molecular background. The first asymmetry was a distinct sidedness, as TROP2 expression maintained its prognostic relevance in right‐sided cancers, but no such association was evident in left‐sided or rectal tumours. In separate analyses between the different UICC stages, a prognostic effect of TROP2 expression was only noted for stage III cancers, but not for stage I, II, or IV tumours. A similar diverging pattern was observed in relation to MSI and p53 status: TROP2 expression demonstrated prognostic significance solely in MSS CRC and those with abnormal p53 expression, with no survival distinctions noted in MSI‐high or p53 wild‐type neoplasms. In contrast to TROP2, conventional histomorphological parameters such as tumour budding or histopathological subtypes (and with the exception of WHO grade) remain prognostically relevant in these subgroups [24] and generally show a higher prognostic impact in univariable analyses.

This assertion gains further support when examining the multivariable analyses that encompass CRC histopathology, molecular determinants, and TROP2 expression. In this context, we note that TROP2 loses its prognostic significance when all these parameters are taken into account. This trend is consistent even in specific subgroups, such as right‐sided tumours, UICC stage III, or MSS/p53‐altered neoplasms, where it initially demonstrated (quite) robust prognostic distinctions in univariable survival analyses. Our study is the first to point out that the prognostic implications of TROP2 are complex and dependent on a variety of covariables, suggesting that it has limited abilities to act as a prognostic biomarker applicable for clinical decision‐making. TROP2 is significantly enriched in a substantial fraction of CRCs exhibiting an invasive histopathological phenotype and other adverse factors, such as advanced tumour stage. Hence, it is highly likely that TROP2 contributes to the aggressive biological behaviour of these tumours. However, many tumours with similar morphological or clinical characteristics do not express TROP2. Despite sharing a comparable phenotype and poor outcome, these tumours appear to rely on alternative TROP2‐independent mechanisms to drive their aggressive biological behaviour. Consequently, it seems logical that the combination of all these parameters, especially tumour budding and stage, surpasses TROP2 as a prognostic parameter.

Our study is subject to certain limitations. The analyses conducted were retrospective in nature, and given the novelty of ADCs in therapeutic oncology, lacked association with TROP2‐directed therapy. Additionally, the study primarily employed TMAs due to the size of our cohort. Nevertheless, we noted a substantial concordance between our TMAs and whole slides, leading us to believe that our data can provide a comprehensive overview of the TROP2 expression landscape in CRC. Moreover, our cohort lacks comprehensive therapy data for each individual patient, making it unfeasible to conduct separate analyses among distinct treatment groups.

In summary, our study reconfirms the quite common occurrence of TROP2 expression in CRC and emphasises its strong correlation with an aggressive phenotype and adverse clinicopathological factors. Future clinical trials exploring anti‐TROP2 ADCs need to consider the observed intratumoural heterogeneity, as only a subset of TROP2‐expressing tumours exhibit strong and uniform positivity. The prognostic implications of TROP2 remain intricate, displaying variations across essential clinicopathological subgroups, suggesting that TROP2 might not be an optimal biomarker for guiding clinical decision‐making.

## Author contributions statement

SF, MS, CD and MJ designed this study. MJ, CD and SF wrote the manuscript with assistance from A‐SL, MT, MS and TG. MJ and A‐SL performed histopathological analysis. MJ, MT, SF and CD performed statistical analyses. MJ, TG, NP, KS and DKB collected clinicopathological data.

## Supporting information


**Figure S1.** Separate univariable survival analyses (log‐rank test) of TROP2 expression in association with DFS in UICC stages I–IV
**Figure S2.** Separate univariable survival analyses (log‐rank test) of TROP2 expression in association with OS in UICC stages I–IV
**Table S1.** Prognostic impact of clinicopathological parameters in the overall cohort
**Table S2.** Frequency and correlations between TROP2 expression and clinicopathological features
**Table S3.** Multivariable survival analyses (DFS) of TROP2 expression in right‐sided CRC including tumour stage as well as histopathological and molecular parameters

## Data Availability

All data relevant for this study are given with the main paper including figures, tables, and the supplemental files. The tissue investigated for this study is archived in the Institute of Pathology of the Technical University of Munich and the Institute of Pathology of the University Hospital Marburg.
