# Supplementary material for: TROP2 in colorectal carcinoma: associations with histopathology, molecular phenotype, and patient prognosis
Source: J Pathol Clin Res. 2024 Aug 23;10(5):e12394. doi: 10.1002/2056-4538.12394 (PMC11342791; doi:10.1002/2056-4538.12394)
Supplement: Supplementary file 1 — Figure S1. Separate univariable survival analyses (log‐rank test) of TROP2 expression in association with DFS in UICC stages I–IV Figure S2. Separate univariable survival analyses (log‐rank test) of TROP2 expression in association with OS in UICC stages I–IV Table S1. Prognostic impact of clinicopathological parameters in the overall cohort Table S2. Frequency and correlations between TROP2 expression and clinicopathological features Table S3. Multivariable survival analyses (DFS) of TROP2 expression in right‐sided CRC including tumour stage as well as histopathological and molecular parameters [file CJP2-10-e12394-s001.pdf]

TROP2 in colorectal carcinoma: associations with histopathology, molecular phenotype, and patient prognosis

S Foersch, M Schmitt *et al*., *J Pathol Clin Res*, https://doi.org/10.1002/2056-4538.12394

Supplementary Figures S1 and S2

Supplementary Tables S1–S3

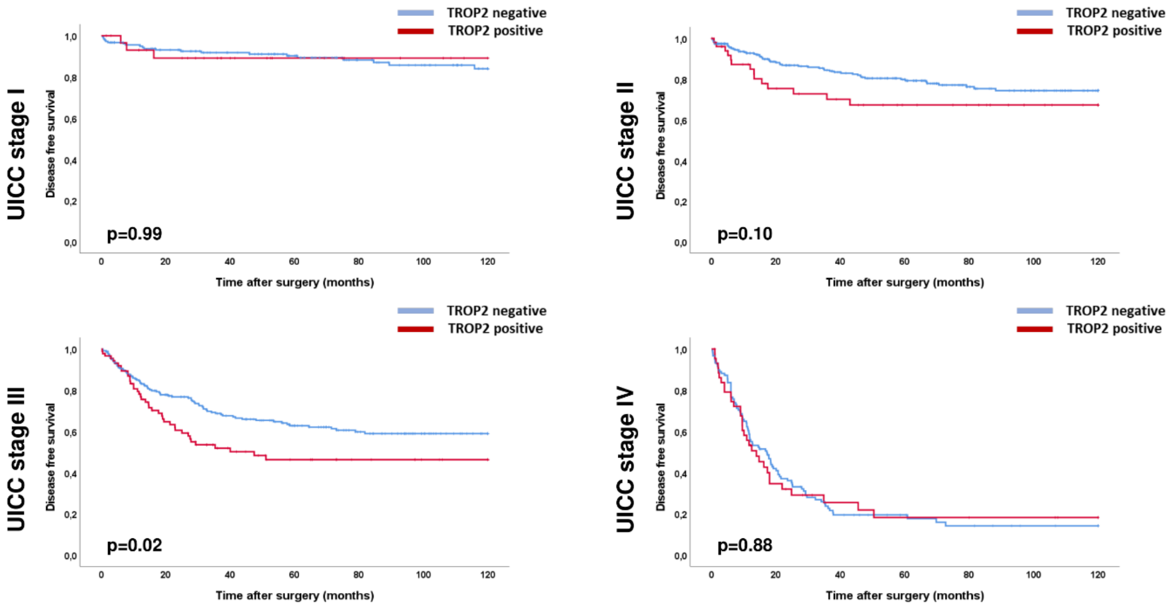

Figure S1. Separate univariable survival analyses (log-rank test) of TROP2 expression in association with disease-free survival in UICC stages I-IV.

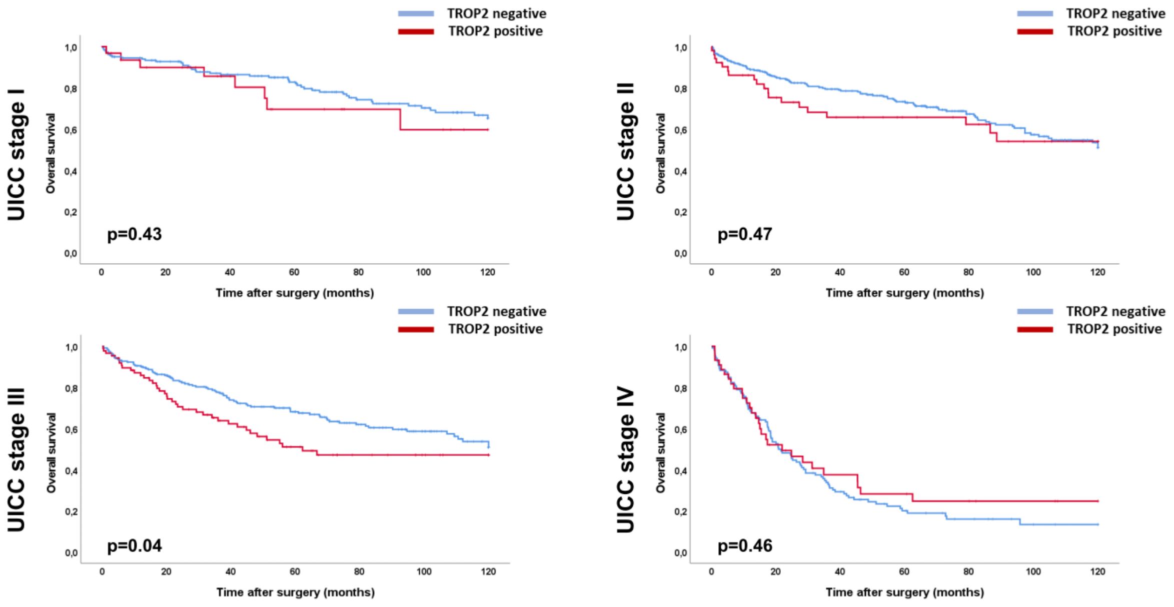

Figure S2. Separate univariable survival analyses (log-rank test) of TROP2 expression in association with overall survival in UICC stages I-IV.

Table S1. Prognostic impact of clinicopathological parameters in the overall cohort.

	Overall n (%)	Mean overall survival (SE) [months]	*p-value*	Mean disease specific survival (SE) [months]	*p-value*	Mean disease free survival (SE) [months]	*p-value*
**Age**			***<0.001***		***0.013***		***0.824***
below median	*509 (48.4%)*	86.41 (2.1)		91.23 (2.1)		82.35 (2.3)	
above median	*543 (51.5%)*	71.62 (2.2)		83.63 (2.3)		81.97 (2.4)	
**Sex**			***0.26***		***0.931***		***0.74***
male	*607 (57.7%)*	77.65 (2.1)		87.47 (2.0)		82.83 (2.2)	
female	*445 (42.3%)*	80.81 (2.4)		87.43 (2.4)		81.14 (2.6)	
**pT**			***<0.001***		***<0.001***		***<0.001***
1	*79 (7.6%)*	97.68 (4.9)		115.54 (2.9)		109.83 (3.6)	
2	*188 (17.9%)*	92.35 (3.2)		103.32 (2.8)		99.37 (3.2)	
3	*587 (55.8%)*	79.56 (2.1)		88.19 (2.1)		81.83 (2.2)	
4	*198 (18.8%)*	56.92 (3.7)		60.15 (3.8)		54.83 (4.0)	
**pN**			***<0.001***		***<0.001***		***<0.001***
0	*581 (55.2%)*	89.00 (1.9)		101.20 (1.7)		99.08 (1.8)	
1	*294 (27.9%)*	75.48 (3.0)		80.75 (3.0)		72.98 (3.2)	
2	*177 (16.8%)*	51.67 (3.9)		54.76 (4.1)		42.97 (3.9)	
**pM**			***<0.001***		***<0.001***		***<0.001***
0	*889 (84.5%)*	86.74 (1.6)		96.61 (1.5)		91.59 (1.7)	
1	163 (15.5%)	39.07 (3.4)		41.56 (3.6)		32.49 (3.4)	
**UICC Stage**			***<0.001***		***<0.001***		***<0.001***
I	*213 (20.2%)*	96.64 (2.9)		111.11 (2.1)		107.84 (2.4)	
II	*351 (33.4%)*	86.01 (2.6)		97.00 (2.4)		95.76 (2.6)	
III	*325 (30.9%)*	80.81 (2.8)		86.68 (2.8)		76.59 (3.0)	
IV	*163 (15.5%)*	39.07 (3.4)		41.60 (3.6)		32.49 (3.4)	
**WHO grade**			***<0.001***		***<0.001***		***<0.001***
Low-grade (formerly G1/G2)	*708 (67.3%)*	86.00 (1.8)		95.24 (1.7)		89.62 (1.9)	
High-grade (formerly G3)	*344 (32.7%)*	65.09 (2.8)		72.13 (2.9)		67.02 (3.0)	
**Resection margins**			***<0.001***		***<0.001***		***<0.001***
R0	*972 (92.4%)*	82.69 (1.6)		92.01 (1.5)		86.86 (1.7)	
R1	*50 (4.8%)*	41.00 (7.2)		42.53 (7.5)		29.47 (6.1)	
R2	*30 (2.9%)*	24.99 (4.5)		25.00 (4.5)		21.52 (3.7)	
**Tumour budding activity**			***<0.001***		***<0.001***		***<0.001***
Bd1 (no/low tumour budding)	*561 (53.3%)*	97.80 (1.7)		109.35 (1.4)		107.01 (1.5)	
Bd2 (intermediate tumour budding)	*271 (25.8%)*	70.31 (3.1)		77.37 (3.2)		66.57 (3.4)	
Bd3 (high tumour budding)	*220 (20.9%)*	41.34 (3.1)		44.27 (3.3)		36.74 (3.3)	
**Histopathological Subtype**			***<0.001***		***<0.001***		***<0.001***
Adenocarcinoma NOS	*651 (61.9%)*	83.53 (1.9)		92.35 (1.9)		87.40 (2.0)	
Mucinous adenocarcinoma	*88 (8.4%)*	76.55 (5.5)		87.03 (5.6)		78.09 (6.0)	
Signet-ring cell carcinoma	*9 (0.9%)*	53.96 (22.5)		54.00 (22.5)		34.42 (18.9)	
Medullary carcinoma	*32 (3.0%)*	98.60 (7.2)		116.32 (3.6)		112.82 (4.9)	
Micropapillary adenocarcinoma	*129 (12.3%)*	53.59 (4.4)		56.21 (4.6)		47.27 (4.5)	
Serrated carcinoma	*91 (8.7%)*	78.40 (5.6)		87.67 (5.4)		84.40 (5.6)	
Adenoma-like adenocarcinoma	*33 (3.1%)*	98.08 (6.4)		115.19 (3.5)		116.43 (3.5)	
MANEC/MiNEN	*10 (1.0%)*	22.41 (7.7)		22.41 (7.7)		20.54 (8.1)	
NEC (SC/LC)	*9 (0.9%)*	40.00 (11.4)		40.00 (11.4)		27.78 (10.3)	
**Tumour localisation**			***0.21***		***0.535***		***0.266***
Right-sided colon	*508 (48.3%)*	76.73 (2.3)		86.58 (2.3)		82.04 (2.4)	
Left-sided colon	*426 (40.5%)*	82.32 (2.4)		89.23 (2.3)		84.16 (2.6)	
Rectum	*118 (11.2%)*	76.43 (4.6)		84.59 (4.7)		75.12 (5.1)	
**MSI Status**			***0.015***		***0.001***		***<0.001***
MSS	*887 (84.3%)*	77.37 (1.7)		85.23 (1.7)		79.55 (1.8)	
MSI	*165 (15.7%)*	88.36 (3.8)		100.88 (3.4)		97.29 (3.7)	
**L**			***<0.001***		***<0.001***		***<0.001***
0	*508 (48.3%)*	89.73 (2.1)		101.79 (1.8)		100.61 (1.9)	
1	*544 (51.7%)*	68.71 (2.3)		73.89 (2.3)		64.91 (2.4)	
**V**			***<0.001***		***<0.001***		***<0.001***
0	*904 (85.9%)*	83.77 (1.6)		93.38 (1.6)		89.24 (1.7)	
1	*148 (14.1%)*	48.88 (4.2)		50.92 (4.3)		39.12 (4.0)	
**Pn**			***<0.001***		***<0.001***		***<0.001***
0	*956 (90.9%)*	81.71 (1.6)		90.92 (1.6)		86.43 (1.7)	
1	*96 (9.1%)*	46.20 (5.4)		47.22 (5.5)		34.32 (4.9)	
**p53 status**			***0.137***		***0.029***		***<0.001***
p53-wildtype	361 (34.3%)	82.04 (2.7)		92.55 (2.6)		91.51 (2.6)	
p53-abnormal	691 (65.7%)	77.39 (2.0)		84.89 (1.9)		77.46 (2.1)	

Table S2. Frequency and correlations between TROP2 expression and clinicopathological features.

		TROP2 negative (IRS 0-1)	TROP2 positive (IRS >2)	*p-value*
**Overall n (%)**	**1052 (100%)**	***838 (79.7%)***	***214 (20.3%)***	
**IRS**	0-1	838 (79.7%)	-	
	2-3	-	51 (4.8%)	
	4-8	-	114 (10.8%)	
	9-12	-	49 (4.7%)	
**Gender**				
	Female	359 (80.7%)	86 (19.3%)	**0.483**
	Male	479 (78.9%)	128 (21.1%)	
**Age**				
	Below median	401 (78.8%)	108 (21.2%)	**0.494**
	Median and above	437 (80.5%)	106 (19.5%)	
**pT**				
	1	68 (86.1%)	11 (13.9%)	
	2	157 (83.5%)	31 (16.5%)	***0.176***
	3	459 (78.2%)	128 (21.8%)	
	4	154 (77.8%)	44 (22.2%)	
**pN**				
	0	495 (85.2%)	86 (14.8%)	***<0.001***
	1	216 (73.5%)	78 (26.5%)	
	2	127 (71.8%)	50 (28.2%)	
**pM**				
	0	719 (80.9%)	170 (19.1%)	***0.022***
	1	119 (73.0%)	44 (27.0%)	
**UICC Stage**				
	I	182 (85.4%)	31 (14.6%)	
	II	300 (85.5%)	51 (14.5%)	***<0.001***
	III	237 (72.9%)	88 (27.1%)	
	IV	119 (73.0%)	44 (27.0%)	
**WHO grade**				
	Low-grade (formerly G1/G2)	577 (81.5%)	131 (18.5%)	***0.033***
	High-grade (formerly G3)	261 (75.9%)	83 (24.1%)	
**Tumour budding activity**				
	Bd1 (No/low tumour budding)	474 (84.5%)	87 (15.5%)	***<0.001***
	Bd2 (intermediate tumour budding)	204 (75.3%)	67 (24.7%)	
	Bd3 (high tumour budding)	160 (72.7%)	60 (27.3%)	
**Tumour localisation**				
	Right-sided colon	404 (79.5%)	104 (20.5%)	***0.032***
	Left-sided colon	350 (82.2%)	76 (17.8%)	
	rectum	84 (71.2%)	34 (28.8%)	
**MSI Status**				
	MSS	689 (77.7%)	198 (22.3%)	***<0.001***
	MSI	149 (90.3%)	16 (9.7%)	
**p53 status**				
	p53-wildtype	308 (85.3%)	53 (14.7%)	***<0.001***
	p53-abnormal	530 (76.7%)	161 (23.3%)	

Table S3. Multivariable survival analyses (disease-free survival) of TROP2 expression in right-sided CRC including tumour stage as well as histopathological and molecular parameters.

		HR (DFS)	lower CI (95%)	upper CI (95%)	p-value
**TROP2 expression**					
	TROP2 negative	1.00			***0.061***
	TROP2 positive	1.45	0.98	2.06	
**Tumour budding activity**					
	Bd1 (no/low tumour budding)	1.00			***<0.001***
	Bd2 (intermediate tumour budding)	3.19	1.98	5.13	
	Bd3 (high tumour budding)	5.31	3.18	8.86	
**Histopathological Subtype**					
	Adenocarcinoma NOS	1.00			***0.078***
	Mucinous adenocarcinoma	1.41	0.81	2.47	
	Singt-ring cell carcinoma	1.85	0.51	6.67	
	Medullary carcinoma	0.36	0.08	1.59	
	Micropapillary adenocarcinoma	1.04	0.65	1.65	
	Serrated carcinoma	1.22	0.73	2.04	
	Adenoma-like adenocarcinoma	0.57	0.08	2.04	
	MANEC/MiNEN	1.87	0.43	8.06	
	NEC (SC/LC)	1.66	0.46	6.02	
**UICC Stage**					
	I	1.00			***0.068***
	II	0.96	0.50	1.84	
	III	1.46	0.65	3.27	
	IV	2.11	0.96	4.63	
**Lymphangiosis**					
	L0	1.00			***0.89***
	L1	0.96	0.50	1.82	
**Blood vessel invasion**					
	V0	1.00			***0.045***
	V1	1.68	1.01	2.81	
**Perineural invasion**					
	Pn0	1.00			***0.466***
	Pn1	1.24	0.70	2.21	
**Resection status**					
	R0	1.00			***0.357***
	R1	1.32	0.73	2.41	
	R2	0.83	0.34	2.02	
**WHO grade**					
	Low-grade (formerly G1/G2)	1.00			***0.238***
	High-grade (formerly G3)	1.24	0.87	1.77	
**Microsatellite status**					
	Microsatellite stable	1.00			***0.231***
	Microsatellite unstable	0.70	0.39	1.25	
**p53 status**					
	Wildtype	1.00			***0.778***
	Aberrant	0.78	0.51	1.19	
**Gender**					
	Female	1.00			***0.838***
	Male	1.04	0.74	1.46	
**Age**					
	Below median	1.00			***0.984***
	Median and above	0.98	0.70	1.37	
